# Interstitial pericytes decrease in aged mouse kidneys

**DOI:** 10.18632/aging.100756

**Published:** 2015-06-05

**Authors:** Ania Stefanska, Diana Eng, Natalya Kaverina, Jeremy S. Duffield, Jeffrey W. Pippin, Peter Rabinovitch, Stuart J. Shankland

**Affiliations:** ^1^ Division of Nephrology, Department of Medicine, University of Washington, Seattle, WA 98104, USA; ^2^ Department of Pathology, University of Washington, Seattle, WA 98195, USA; ^3^ Biogen Idec, Cambridge, MA 02142, USA

**Keywords:** endothelium, NG2, PDGFß-receptor, nephropathy, tubulo-interstitial fibrosis

## Abstract

With increasing age, the kidney undergoes characteristic changes in the glomerular and tubulo-interstitial compartments, which are ultimately accompanied by reduced kidney function. Studies have shown age-related loss of peritubular vessels. Normal peritubular vessel tone, function and survival depend on neighboring pericytes. Pericyte detachment leads to vascular damage, which can be accompanied by their differentiation to fibroblasts and myofibroblasts, a state that favors matrix production. To better understand the fate of pericytes in the aged kidney, 27 month-old mice were studied. Compared to 3 month-old young adult mice, aged kidneys showed a substantial decrease in capillaries, identified by CD31 staining, in both cortex and medulla. This was accompanied by a marked decrease in surrounding NG2^+^/PDGFRß^+^ pericytes. This decrease was more pronounced in the medulla. Capillaries devoid of pericytes were typically dilated in aged mice. Aged kidneys were also characterized by interstitial fibrosis due to increased collagen-I and -III staining. This was accompanied by an increase in the number of pericytes that acquired a pro-fibrotic phenotype, identified by increased PDGFRß^+^/αSMA^+^ staining. These findings are consistent with the decline in kidney interstitial pericytes as a critical step in the development of changes to the peritubular vasculature with aging, and accompanying fibrosis.

## INTRODUCTION

During kidney aging, several predictable and characteristic structural changes affect the vast majority of the increasing elderly population. These include glomerular scarring (glomerulosclerosis) affecting part (segmental) or all (global) of individual glomeruli, tubular atrophy, interstitial fibrosis and arteriosclerosis [1, 2]. Consequently, several terms have been used to describe this histological constellation including “nephrosclerosis of aging,” “senile nephrosclerosis” or “aging nephropathy”. It is noteworthy that these are precisely the histological changes seen in patients with chronic kidney disease (CKD) of any age. Indeed, age is a strong predictor of CKD, present amongst 47% of adults ages 70 years and older, compared to 4% of adults ages 20–39. A lack of data fuels continual debates whether age-related histological changes in the kidney are responsible for the inevitable decline in glomerular filtration rate (GFR), or whether the changes are “senile” versus a “disease state” [3]. Kidney outcomes are substantially worse in older patients than younger patients with similar diseases [4]. Moreover, the aging kidney has reduced capacity to repair; for example the proliferative potential of epithelial cells in response to injury is reduced and the tendency for apoptosis is increased [5]. Aged kidneys exhibit higher susceptibility to toxic or ischemic insults, and aging is associated with chronic inflammation and increased oxidative stress [6].

Several studies have shown a reduced or rarefied microvasculature, and morphological changes to endothelial cells in aging, supporting the notion that vascular damage plays a central role in progression of fibrosis in aging [7, 8] There is evidence of impaired angiogenesis and endothelial dysfunction in the aging kidney which is of crucial importance in cases of ischemia and subsequent tissue regeneration [9]. There is a reduction in renal blood flow in aging, and aged kidneys have enhanced response to vasoconstrictors, and diminished response to vasodilators such as nitric oxide (reviewed in: [10]. Focal loss of peritubular capillaries has been shown in the aging rat in the areas of tubulointerstitial fibrosis [11]. A loss of peritubular capillaries in aging rats is correlated with alterations in vascular endothelial growth factor (VEGF) and thrombospondin-1 (TSP-1) expressions. Pro-angiogenic VEGF level is decreased in the medulla, whereas anti-angiogenic TSP-1 is upregulated in the glomeruli and tubulointerstitium [12]. Preservation of microvascular density following ischemia seems to be a key mechanism protecting the kidney from vascular damage [13]. Activation of hypoxia inducible factor 1 (HIF-1) together with HIF-regulated genes VEGF and glucose transporter-1 (GLUT-1) have been found in the aging rat. The most prominent site of HIF-1 expression was in cortical tubules [14].

Pericytes are normally embedded in the vascular basement membrane together with endothelial cells [15, 16]. Pericytes and endothelial cells through the shared basement membrane make numerous contacts such as peg-socket, adhesion plaques and gap junctions, however incomplete basement membrane coverage has also been described [17]. The intense communication between these two cell types takes place. Pericyte functions include maintenance of vascular homeostasis, stabilization of the vascular wall, blood pressure and vascular tone regulation, secretion of growth factors, and modulation of the extracellular matrix [18]. Identification of pericytes by electron microscopy is not practical, therefore a combined criteria of their location, morphology and protein expression is used for identification [18]. Pericytes express certain mesenchymal and neurotrophic markers, including platelet-derived growth factor receptors (PDGFRβ, PDGFRα), as well as neural/glial antigen 2 (NG2), CD146, RGS5, and P75NGFR [19]. It is important to note that none of these markers are specific and protein expression in pericytes differs depending on the tissue, developmental stage, and disease state [18]. Pericytes are also a local source of progenitor cells. In their native perivascular location, they share surface markers with mesenchymal stem cells (MSC) [20]. Similar to MSC, primary pericytes in culture can be differentiated into adipocytes, osteoblasts and chondrocytes [20]. Tri-lineage differentiation ability is not shared by fibroblasts, an interstitial cell type producing collagen [21]. Finally, pericytes can be induced to vascular smooth muscle cell (VSMC) fate following treatment with TGFβ [22] or Ang-II [23]. There is a controversy regarding the interrelationship between pericytes and VSMC [24], because some pericyte populations, such as those in the vasa recta, express alpha smooth muscle actin (αSMA) and are contractile [25, 26]. Interestingly, a study by Dar et al. [27], suggests that pericytes derived from human pluripotent cells characterized by CD146, NG2 and PDGFRβ expression, but not αSMA are more primitive vascular progenitors. Paradoxically, in the context of kidney injury, pericytes may also play a pathological role. Following certain insults, pericytes detach, and migrate away from vessels where they differentiate into myofibroblasts (activated fibroblasts), the main cell type contributing to scar formation [28–30].

To our knowledge, the pericyte has not been investigated in the aged kidney, and the interstitial changes of advanced age are not well defined in mice. We hypothesized that pericytes, *via* different mechanisms such as loss of physiological function, decreased regenerative capacity, and/or pathological transition into myofibroblasts, may contribute to deterioration of kidney function in aging nephropathy.

## RESULTS

### 27-month old female C57BL/6 mice show interstitial fibrosis

27 month old mice are equivalent to a human age of 75 years [31]. The body weights increased in aged mice (23.82 ± 0.42 vs. 26.14 ± 0.77 g, p=0.0402 vs. young adult mice). To monitor kidney function, the albumin-to creatinine ratio (ACR) was measured on spot urines. Aged mice had significantly elevated ACR (84.8±18.49 vs. 22.76±2.99, P=0.0032 vs. young mice).

**Figure 1:**
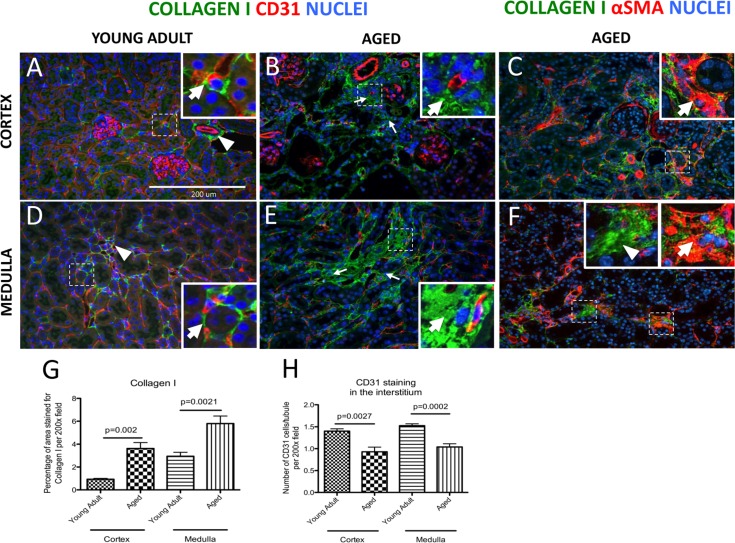
Increase in interstitial fibrosis and reduction of microvascular density in aged mice kidneys Fibrosis was measured by staining for collagen I (green color). Double staining of αSMA (intracellular antigen) and collagen I was used to discern collagen I-expressing myofibroblasts from extracellular matrix proteins. Microvascular rarefaction was assessed by CD31 staining (red). DAPI staining identifies nuclei (blue). (**A**) Collagen I staining was faint and confined to the fibroblasts/pericytes in the cortex of young adult kidneys. The inset shows a high power image of a collagen I^+^ cell (green) encircling capillary (red) (arrow). Adventitial cells of renal arteries also stain for collagen I (arrowhead). (**B**) Collagen I staining was more abundant in the cortical interstitium of aged kidneys (arrows indicate examples) and was found outside the capillary walls (inset, arrow shows collagen I staining). CD31 staining was reduced in intensity. (**C**) Co-staining for collagen I and αSMA shows the presence of myofibroblasts (inset, arrow). (**D**) Collagen I staining in the medulla of young adult mice was present in vasa recta (arrowhead) and perivascular fibroblasts (inset, arrow). (E) In aged kidneys interstitial collagen I staining was increased (arrows indicate examples). The inset shows high power view of accumulation of collagen I staining (arrow). CD31 staining was decreased. (**F**) Co-staining of collagen I with αSMA confirms the presence of myofibroblasts (inset, arrow). Extracellular collagen I was also detected (inset, arrowhead). (**G**) Graph showing quantification of interstitial collagen I staining was significantly increased in both the cortex and the medulla. (**H**) Quantification of CD31 staining shows reduced vascular density per tubule in both the cortex and the medulla. Data are represented as mean ± SEM (n=6).

To visualize changes in fibrosis in the kidney interstitium, collagen-I staining was performed. Perivascular cells, including pericytes and perivascular fibroblasts, express collagen-I [29]. By definition, pericytes are embedded in the same basement membrane with endothelial cells whereas fibroblasts are embedded in extracellular matrix [19]. In young adult mouse cortex, perivascular cells of peritubular capillaries (Figure 1A, arrow), and the adventitial cells of arteries (arrowhead, Figure 1A) were positive for collagen-I staining. In aged mice, an expansion of collagen-I positive areas was observed in the interstitium (arrows, Figure 1B). Collagen 1 expressing cells were identified as myofibroblasts by co-staining for αSMA (arrows, Figure 1C). Collagen-I protein staining was not detected in glomeruli (Figure 1A-B).

In the medulla of young adult mice, occasional collagen-I producing cells were located along peritubular capillaries (arrow, Figure 1D) and in the vasa recta (arrowhead, Figure 1D). In the aged kidney medulla, deposition of collagen-I was greatly increased in the interstitial spaces (arrows, Figure 1E). The intracellular location of collagen I was confirmed by αSMA co-expression (arrow, Figure 1F). However, to some extent collagen 1 staining was clearly extracellular, when DAPI was used to identify nuclei (arrowhead, Figure 1F). When quantitated, the percentage of area stained for collagen I was significantly increased in the cortex of aged mice (0.93±0.07% vs. 3.62±0.52%, young adult vs. aged, p=0.002), and in the medulla of aged mice (2.95±0.34% vs. 5.79±0.66%, young adult vs. aged, p=0.0021) (Figure 1G).

**Figure 2:**
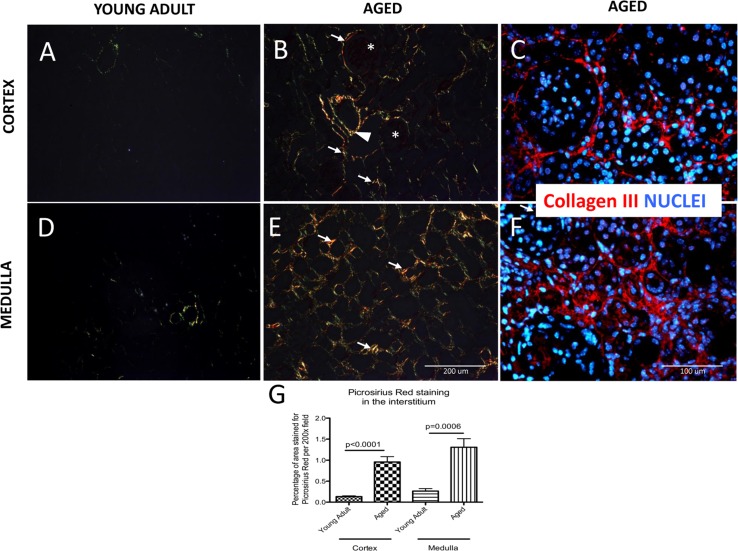
Accumulation of type I and III collagen in aged kidneys Picrosirius Red staining of collagen I and III was examined by polarized light microscopy. Additionally, collagen III fibers are visualized with specific antibody (red color), nuclei are labeled with dapi (blue color). (**A**) Collagen fibers were sparse in the cortex of young adult kidneys. (**B**) Aged kidneys demonstrated interstitial collagen deposition (arrows indicate examples) in the peritubular and periglomerular area (glomeruli are indicated by asterix). Adventitia of arterial wall was enriched in collagen (arrowhead). (**C**) Collagen III staining confirms the location of interstitial and periglomerular fibrosis. (**D**) Picrosirius Red was barely detected in the medulla of young adult kidneys. (**E**) Picrosirius Red staining was markedly increased in the medulla of aged mice (arrows indicate examples) in the interstitium. (**F**) Collagen III staining confirms tubulointerstital fibrosis. (**G**) Graph of quantitation: Picrosirius Red staining significantly increased in the interstitium in both the cortex and the medulla of aged mice. Data are represented as mean ± SEM (n=6).

Additionally, Picrosirius Red staining for collagens I and III [31] was performed. Polarized light microscopy was used to visualize and distinguish the thick, strongly birefringent, yellow/red fibers as collagen type I and the thin, weak birefringent, greenish fibers as collagen III [32]. In young adult mouse kidneys, Picrosirius Red staining was very faint. Occasional fibers of collagen III were detected (Figure 2A). In aged kidneys, Picrosirius Red positive staining was increased in the interstitium (i.e. between tubules), and periglomerular areas. In contrast, Picrosirius red staining was not detected within glomeruli (asterix, Figure 2B). Additionally, collagen fibers were present in the adventitia of large blood vessels (arrowhead, Figure 2B). Collagen III immuno-staining confirmed that tubulointerstitial and periglo-merular fibrosis characteristic of the aged kidney (Figure 2C). The percentage of area stained for Picrosirius Red was significantly higher in the aged kidney cortex compared to young adult mice (0.96±0.13% vs. 0.13±0.02% p<0.0001) (arrows, Figure 2, B and G).

Similarly, in the medulla of young adult mice staining for collagen fibers was scarce (Figure 2D) whereas in the aged kidney medulla there was an increase in collagen deposition in the interstitium (arrows, Figure 2E). Using the colors on polarizing microscopy to differentiate between collagen I and III, our results showed that both collagen I (yellow and red fibers) and collagen III (green fibers) were increased in the aged kidney compared to the young adult kidneys. Collagen III immunostaining confirmed the tubulointerstital location of fibrosis in the aged kidney (Figure 2F). The percentage of the interstitial area stained for Picrosirius Red in the medulla was higher in aged mice (1.31±0.20% vs. 0.26±0.06%, p=0.0006 aged d vs.

young adult mice) (arrows, Figure 2E, G). Taken together, these results show that interstitial fibrosis was increased in the cortex and medulla of aged mice compared to young adult mice.

### Interstitial vascular density declines in the aged kidneys

To assess changes in vascular density in aging, CD31 staining was used to label endothelial cells and the number of CD31 positive cells per tubule was quantified in both cortex and medulla (Figure 1H). In the cortex, microvascular density was decreased from 1.40±0.05 in young adult mice to 0.93 ± 0.11 in aged mice (p = 0.0027). In the medulla, microvascular rarefaction was even more pronounced (1.52 ± 0.04 vs.1.04 ± 0.07, young adult vs. aged, p < 0.0002) (Figure 1H). In aged mice compared to young adults, there was a reduction in CD31 staining in the interstitium (Figure 1A-B and 1D-E). Taken together, 27 month old mouse kidneys represent a robust model of kidney aging.

### Decrease in the number of interstitial pericytes in the aged kidney

**Figure 3:**
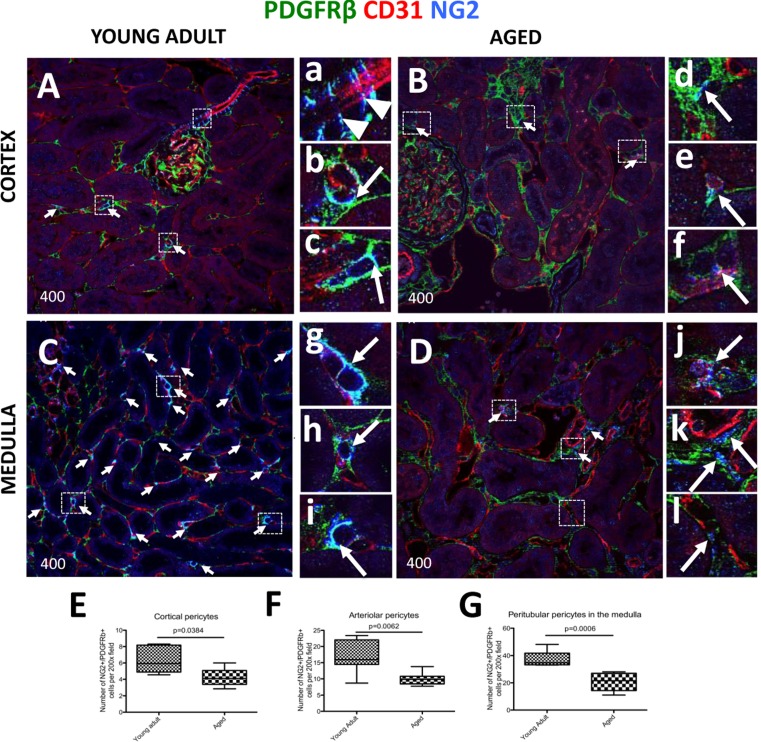
Decreased expression of kidney pericyte markers in aged mice Pericytes were identified by double NG2^+^/PDGFRß^+^ staining (blue and green colors respectively), and their perivascular location was identified by endothelial CD31 staining (red color). Double positive cells (light blue color) were quantified using single channel images (marked here by the arrows). (**A**) In young adult kidney cortex, staining for pericyte markers was readily detected alongside afferent arterioles (a, arrowheads) and peritubular capillaries (examples indicated in the boxed regions b and c, arrows). (**B**) In aged kidney cortex, the number of cells staining for pericyte markers and lining interstitial peritubular capillaries was reduced. Pericytes surrounding the vessels show loss of contacts with endothelial cells (d, arrow), and CD31 staining intensity was reduced (e, f, arrows). (**C**) Kidney medulla of young adult mice showed abundant pericytes located along peritubular capillaries. The boxed regions show higher power images of pericytes tightly encircling peritubular capillaries (g, h, i, arrows). (**D**) In aged kidney medulla, there was a decline in number of pericytes surrounding peritubular capillaries. Pericyte staining intensity diminished (examples are shown in the boxed regions j, k, l arrows), and the vessel coverage was lacking (k,l, arrows). In aged mice compared to young adults, pericyte number was decreased in: (**E**) peritubular capillaries in the cortex, (**F**) pre-capillary arterioles, (G) peritubular capillaries in the medulla. Data are represented as mean ± SEM (n=6).

In the non-glomerular areas of the tubulointerstititum, several populations can be classified as pericytes: interstitial pericytes surrounding peritubular capillaries [33], vasa recta pericytes [34], and arteriolar pericytes [35]. In the current study, pericytes were identified based on, and required, the following three criteria: (i) cells staining for both PDGFRß and NG2 (PDGFRß^+^/NG2^+^), (ii) located in a perivascular niche, and (iii) encircling CD31^+^ endothelial cells. All the figures in the results section used confocal microscopy. As expected, in young adult kidney cortex, pericyte markers labeled afferent arterioles (arrowheads, Figure 3A, a), and peritubular capillaries (arrows, Figure 3A, b-c). However, in the aged kidney, there is a marked decrease in pericyte staining in peritubular capillaries (arrows, Figure 3B, d-f). In young adult kidney medulla, pericytes were found in abundance, especially in the outer medulla where they closely surround peritubular capillaries (arrows, Figure 3C, g-i). In contrast, in aged kidneys there was a marked decrease in pericyte marker staining and intensity (arrows, Figure 3D, k-l). Moreover, aged pericytes do not completely cover endothelial cells, consistent with a disruption in pericyte-endothelial cell interaction (arrows, Figure 3D, k-l).

Quantitation showed a decline in overall pericyte number in the interstitium of aged kidneys compared to young adult kidneys (Figure 3E-G). The results based on pericyte location were as follows: peritubular capillary pericyte number in the cortex was reduced in aged mice (4.20±0.51 vs. 6.31 ± 0.67 per field of view, p = 0.0384 vs. young adult mice) (Figure 3E). In pre-capillary arterioles, pericyte number also decreased in aged kidneys (10.05±0.73 vs. 16.66±1.856 per field of view, p < 0.0062 vs. young adult mice) (Figure 3F). Pericyte number associated with medullary peritubular capillaries was greatly reduced in aged kidneys (37.48 ± 2.14 vs. 20.74 ± 2.83, p < 0.006 vs. young adult) (Figure 3G). Although the number of pericytes covering vasa recta in the medulla was greatly reduced, it did not reach statistical significance (15.05±1.70 vs.10.17±2.12, young adult vs. aged) (not shown). Overall, the aged kidney had a reduced number of pericytes, in which the most affected area was the medulla.

**Figure 4:**
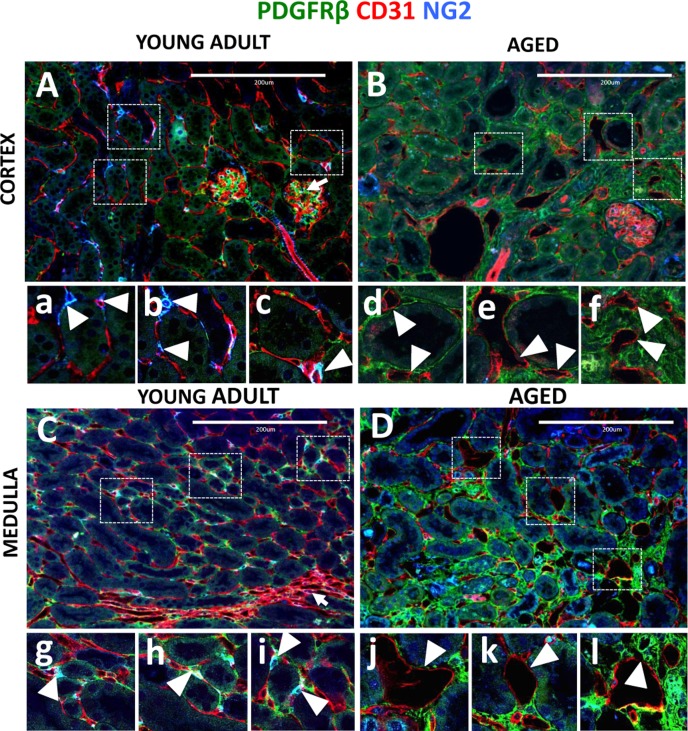
Capillary dilation in aged mice was associated with reduced pericyte coverage Endothelial cells were marked by CD31 expression (red color). Pericytes were labelled by NG2^+^/PDGFRß^+^ staining (blue and green colors respectively). Double positive cells (light blue) were quantified using single channel images. (**A**) In young adult kidney cortex, peritubular capillaries form a regular pattern around renal tubules and inside the glomerular tuft (arrow). The boxed regions show higher power images of interstitial pericytes supporting peritubular capillaries (a, b, c, arrowheads). (**B**) In aged kidney cortex, endothelial cells become dilated, have decreased pericyte coverage (d, e, arrowheads) and some were completely isolated from tubules (f, arrowheads). (**C**) In young adult medulla, endothelial cells occupy peritubular spaces and vasa recta (arrow). Typically, few capillaries surround each tubule and pericyte, and are closely attached to the endothelial cells (g, h, i, arrowheads). (**D**) In aged kidney medulla, dilated peritubular capillaries were also present (j, k, arrowheads). In some instances, dilated capillaries appear in the areas of increased interstitial PDGFRß+ staining (l, arrowhead).

In both the cortex (arrowheads, Figure 4A, a-c) and medulla (arrowheads, Figure 4C, g-i) of young adult kidneys, CD31^+^ endothelial cells formed a dense network, with several capillaries wrapped around each tubule. In aging, in addition to decreased vascular density (Figure 1F), capillary dilation was observed in the cortex (Figure 4B), and medulla (Figure 4D). Higher magnification images show that dilated vessels typically lacked pericyte support, had thinned capillary walls and abnormal shape (arrowheads, Figure 4B, d-f and 4D, j-l). Moreover, some of the capillaries were separated from tubules and are completely embedded in the interstitium (arrowheads, Figure 4B, f and 4D, l).

### Pericytes were profibrotic in aging nephropathy by differentiating in to myofibroblasts

**Figure 5:**
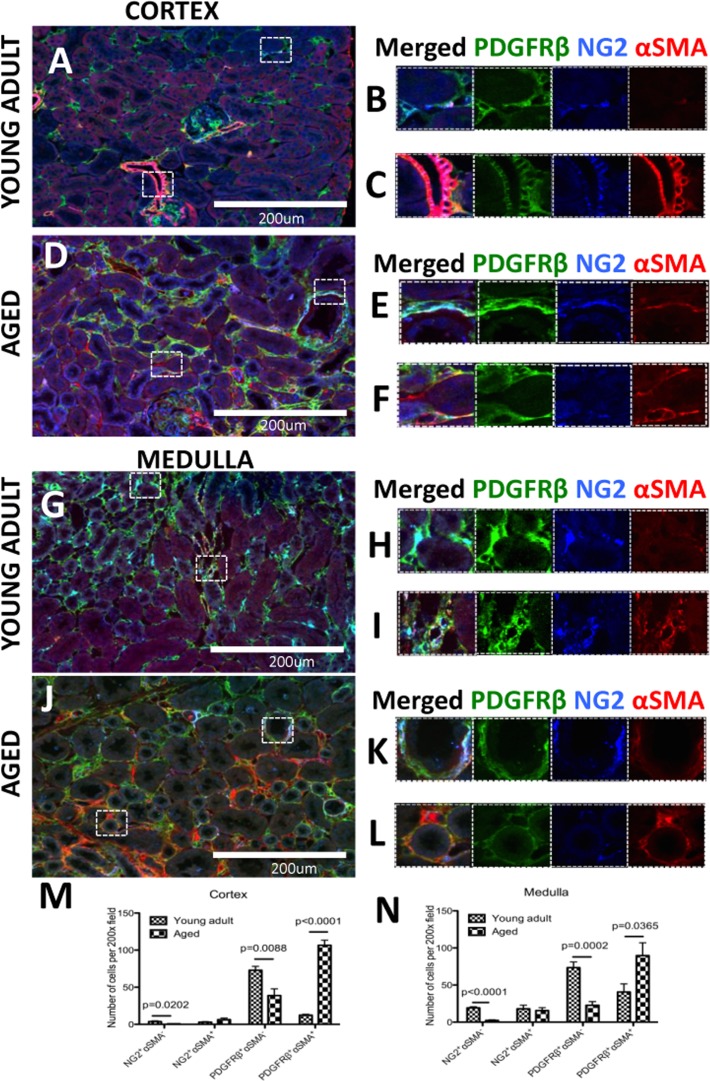
A subset of pericytes differentiate into myofibroblasts and increase in aged kidneys Pericytes were identified by NG2^+^/PDGFRß^+^ staining (blue and green colors respectively). αSMA was used as myofibroblast marker (red color). (**A**) In the young adult kidney cortex, (**B**) most pericytes do not express αSMA. However in preglomerular arterioles (**C**) αSMA expression was present together with NG2/PDGFRß staining. (**D**) In aged kidney, αSMA increased in pericytes (**E**) and PDGFRß^+^ /NG2^−^ cells (**F**). (**G**) Medulla of young adult kidney showed the presence αSMA in some peritubular capillaries (**H**) and contractile vasa recta cells (**I**). In aged mouse kidney, (**J**) there was accumulation of αSMA^+^ cells co-expressing PDGFRß and NG2 (**K**) or PDGFRß only (**L**). Quantification of αSMA expression in NG2^+^ and PDGFRß ^+^ cells showed that PDGFRß^+^ cells outnumber NG2^+^ cells in both young adult and aged kidneys. There was a dramatic increase in PDGFRß^+^αSMA^+^ cells and the numbers of αSMA-cells expressing either PDGFRß or NG2 were significantly lower in both (**M**) the cortex and (**N**) the medulla. Data are represented as mean ± SEM (n=6).

Perivascular cells are considered a possible source of kidney fibrosis in chronic kidney injury [28, 29]. In order to determine the subset of pericytes that differentiate into myofibroblast in the aged kidney, pericytes (identified by PDGFRß**^+^,** NG2**^+^**) were co-stained for αSMA to mark myofibroblasts [36] In young adult kidney cortex (Figure 5A), αSMA staining is occasionally detected in peritubular pericytes (Figure 5B), and always in vascular smooth cells of renal arterioles (Figure 5C). In aged kidney cortex (Figure 5, D), there was an increase of αSMA-expressing pericytes/perivascular cells in regions of tubulointerstitial fibrosis (Figure 5E-F). In the young adult medulla (Figure 5G), most pericytes did not express αSMA (Figure 5H), with the exception of the vasa recta (Figure 5I). In aged kidney, αSMA staining increased substantially (Figure 5J). Overlapping staining for αSMA and pericyte markers was confined to tubulointerstitial fibrosis (Figure 5K-L).

Quantification shows that the majority of αSMA-expressing cells share PDGFRß+ marker expression (Figure 5M-N). In the kidney cortex of aged mice (Figure 5M), there was a large increase in the number of PDGFRß^+^/αSMA**^+^** cells (12.29±1.33 vs.106.04±6.9,

young adult vs. aged, p<0.0001). This was accompanied by a decrease in PDGFRß^+^/αSMA^−^ cells (72.88±5.34 vs. 38.84±9.02, young adult vs. aged, p<0.0088) which might suggest a transition of these cells towards αSMA+ myofibroblasts. However, fate-mapping studies will be required to confirm this. There was a slight increase of NG2^+^ /αSMA^+^ cells (2.99±0.67 vs.6.36±2.12, young adult vs. aged), which was not significant. This was also accompanied by a decrease in the NG2^+^/αSMA^−^ cell population (3.66±1.05 vs. 0.65±0.28, young adult vs. aged, p=0.0202). However it appeared that the overall number of NG2^+^ populations were much smaller.

In the kidney medulla of aged mice (Figure 5N), the number of NG2^+^/αSMA^−^ cells was lower compared to young adult mice (19.15±1.48 vs. 2.26±0.88, p<0.001), as was the number of PDGFRß^+^ /αSMA^−^ cells (73.44±7.63 vs. 22.74±4.98, young adult vs. aged, p<0.0002). As with the cortex, the number of NG2^+^/αSMA^+^ cells did not change significantly with age (15.99±5.74 vs. 15.56±3.86, young adult vs. aged). However, the number of PDGFRß^+^/αSMA^+^ cells was higher in aged kidneys (40.59±10.93 vs. 91.08±17.85, young adult vs. aged, p=0.05).

## DISCUSSION

Pericyte depletion has been implicated in the pathology of several chronic diseases such as diabetic retinopathy [37], cancer progression [38], and Alzheimer's disease [39]. Alterations in pericyte-endothelial cell interactions underlie microvascular complications [40]. To our knowledge, the current study is the first to report a decline in pericyte number in aged kidneys. We show that age-related pericyte loss is linked to impaired pericyte coverage of blood vessels in the interstitium and pre-capillary arterioles. We also observed an accumulation of pericytes co-expressing αSMA, suggesting differentiation of these cells into myofibroblasts with advanced age.

Although the morphological changes in aged rat [11, 12] and human [41] kidneys have been well characterized, most studies in mice, including our previous report [42], have been limited to middle-aged mice. Twenty seven-month old mice were investigated, which are considered advanced age (defined by the National Institute of Aging), equivalent to a human aged 75 years [43, 44]. To our knowledge, this is the first description showing kidney scarring and reduced vascularity in such old mice kidneys. These aged mice therefore are quite representative of their human counterparts, and therefore serve as a representative model to better understand age-related kidney events. Mouse models have been extensively used in aging research [45, 46]. In particular in the kidney, similar physiological changes have been observed in mouse and human 47, 48].

Having shown significant changes in the interstitial vasculature, we next focused on pericytes. The rationale follows that pericytes perform a key biological role in providing survival factors for endothelial cells, and are critical for the overall integrity of the underlying vessels around which they are typically wrapped, and may be a source of mesenchymal stem cells [15, 24]. Pericytes have been extensively described in normal adult human and mouse kidneys [17, 33, 49]. Although several markers have been used to identify pericytes, we used co-expression of PDGFRß and NG2 to identify a major population of pericytes [20, 29]. The first major finding was that the absolute number of interstitial kidney pericytes was significantly lower in aged mouse kidneys compared to young kidneys. Moreover, the decrease was more pronounced in the medulla of aged mice compared to the cortex. Noteworthy was the close correlation in aged kidneys between the lower pericyte number and the following two changes in underlying peritubular microvessels. First, there was a loss of the endothelial cell marker CD31, consistent with rarefaction. Second, peritubular vessels devoid of pericytes were often dilated. It is interesting to note that when pericyte function was disrupted during nephrogenesis by targeting Foxd1 transcription factor, this also resulted in abnormal vascular patterning and dilated capillaries [50]. Similarly, PDGFß knockout mice present a phenotype lacking microvascular pericytes and are characterized by capillary micro-aneurysms [51]. Pericytes do not only regulate capillary integrity, but also the size of microvessels due to regulation of vessel constriction and relaxation. In some instances pericytes respond to pathological insults by prolonged microvessel constriction that may eventually lead to pericyte death [52, 53]. In several diseases such as Alzheimer's disease, cerebral ischemia, and diabetic retinopathy, pericyte loss has been associated with ß-amyloid, hypoxia and high glucose respectively (reviewed in:[54, 55]). Preservation of capillaries is of great importance in kidney disease; normalization of vasculature and restoration of pericytes could prevent both hypoxia and fibrosis [56].

The mechanisms underlying peritubular vascular changes in the aged kidney are not well characterized. Although these studies are descriptive, we speculate that the possible course of events with advanced age, is a vascular survival or angiogenic factor imbalance secondary to reduced pericyte number triggers endothelial cell proliferation and pericyte migration from vessels. In chronic disease the balance of angiogenic of vascular survival factors shifts towards anti-angiogenic, leading to pericyte detachment and endothelial cell dysfunction and apoptosis (reviewed in: [57]).

The mechanisms that maintain normal pericyte number in adult kidneys are not well delineated. In aging, reparative mechanisms are impaired and this could be due to stem cell loss in aging [58, 59]. Age-related changes in stem cells are manifested by loss of lineage specificity, failure self-renewal or/and senescence. A recent study indicates a subpopulation of kidney pericytes are MSC [60]. Since pericytes derive from MSC, it is possible that pericyte loss may reflect MSC senescence. Additionally, the local environment of the stem cell “niche” may become progressively disrupted through a failure to provide trophic factors and spatial support [61]. Furthermore, changes to vasculature within stem cell niche may also modulate stem cell function [62].

The decline in kidney function resulting from interstitial and glomerular fibrosis has been well characterized in aging [63]. There has been a great interest in revealing the ultimate identity of any fibrosis-producing cells. Although pericytes are important in nephrogenesis, homeostasis and regeneration, pericytes can paradoxically directly promote fibrosis [28, 29]. Pericytes are stromal-derived cells, and can therefore be identified in part by PDGFRß expression. However, there is no single marker to differentiate the pro-fibrotic subset of pericytes from fibroblasts or even vascular smooth muscle cells [36, 64]. A second major finding of this study was that the number of stromal cells of PDGFRß that co-expressed αSMA, but not NG2, was substantially higher in aged kidneys. PDGFRß^+^ αSMA^+^ co-localized to areas of interstitial fibrosis. LeBleu [65] reported a contribution of PDGFRß+ derived cells to kidney fibrosis and showed, in agreement with results presented here, that PDGFRß^+^NG2^−^-expressing cells were the key mediators in promoting fibrosis compared to PDGFRß+NG2+ cells. Blocking of PDGFRß signaling in kidney fibrosis resulted in reduction of pericyte activation and subsequent differentiation into myo-fibroblasts [66, 67]. Given that NG2-pericytes are well described, it is likely that many of the PDGFRβ^+^NG2^−^ cells are a distinct pericyte subpopulation, but at this time there is no marker to separate them from resident fibroblasts. Although proving this is beyond the scope of the manuscript, we think that the data makes a compelling story that in aged kidneys, pericyte transdifferentiation might be one of the causal factors underlying kidney fibrosis.

It is worth mentioning when comparing aging with inducible disease models, that the timescale and disease acuity are very different from aging. In models of kidney disease, usually young mice undergo 1–2 weeks of disease induction, whereas aging involves much longer period of disease progression with repair mechanisms very likely disabled. In the interstitium of aged kidneys, the number of pericytes decrease, yet the number of NG2-pericytes that have differentiated into myofibro-blasts increases. Therefore our results are in keeping with a role for pericytes as a myofibroblast precursor cell in the aged kidney and suggest pericytes are a central cell that directly contributes to the decrease in peritubular vasculature and increase in interstitial fibrosis.

In summary, this study highlights the potential role of reduced kidney interstitial pericyte number in the age-related decrease in peritubular capillaries, and the accompanying increase in interstitial fibrosis. Further studies on mechanisms underlying reduced kidney pericyte number in aging are needed to define pathways that lead to fibrosis.

## METHODS

### Animals

Aging nephropathy was studied in C57BL/6 female mice obtained from the National Institutes of Aging. Mice were housed in standard conditions with access to food and water *ad libitum*. Aged mice were euthanized at age 27 months (n=7); mice aged 3 months old (n=7) served as controls. Kidneys were harvested and fixed in 10% buffered formalin for histology. All experiments were carried out in compliance with the rules established by the University of Washington Animal Care and Use Committees.

### Kidney function

Mouse albumin in urine was measured by radial immunodiffusion assay (RID) as previously described [68]. Briefly, rabbit anti-mouse albumin antibody (Accurate Chemical, Westbury, NY) and rabbit serum (Pel-Freez, Rogers, AR) were incorporated into a thin layer of 1.5% type I, low EEO agarose gel (Sigma-Aldrich) in 0.5 M veronal buffer. A small volume of urine was placed in a well cut into the agarose gel. While urine sample diffused from the well, specific anti-albumin antibody reacted in the agar forming a halo of precipitation around the well. After the antibody reaction reached saturation, the diameter of the halo was measured. The size of the halo was related directly to the albumin concentration in the urine based on standard curve prepared from known concentrations of purified fraction V mouse albumin standards (MP Biomedicals, Irvine, CA). Creatinine was measured in the urine via a colorimetric assay (Cayman Chemical, Ann Arbor, MI) and an albumin to creatinine ratio was calculated.

### Fibrosis scoring

Histological analysis of fibrosis was carried out on fixed renal tissue, embedded in paraffin, and sectioned at a thickness of 4 μm. Connective tissue deposition was examined with Picrosirius Red Stain Kit (Polysciences, Inc, Warrington, PA, USA) and collagen I (1:100, Millipore, Billerica, MA, USA), staining, as we have previously described [69-71]. Picrosirius Red-stained sections were photographed under polarized light to achieve maximal brightness and the percentage of positive interstitial staining was quantified. For collagen I staining fluorescent images were collected with Leica DFC310 FX camera. At least 10 images of both cortex and medulla were analyzed. *Image J* image analysis software was used to quantify the percentage of tissue fibrosis in the cortex and medulla [69-71]. Greyscale images were subjected to threshold analysis producing the value of pixel intensity representing positive staining and then expressed as a percentage of the value of pixel intensity of the whole image. Additionally, anti-collagen III (1:100; Abcam, Cambridge, MA, USA) staining and double staining for collagen I and αSMA (1:10.000; Sigma, Saint Louis, MI, USA) was performed to assess specifically contribution of different types of collagen to fibrosis in a qualitative manner.

### Pericyte assessment

NG2^+^ pericytes were identified by the co-expression of PDGFRß and NG2 antigens, their perivascular location and morphology [33, 49]. Formalin-fixed kidney sections underwent deparaffinization, heat-mediated antigen retrieval in citrate buffer pH 6.0, and blocking unspecific background (Accurate, San Jose, CA, USA). Avidin/biotin blocking (Vector Laboratories, Burlingame, CA, USA) was performed to block endogenous biotin and prevent unspecific staining while using biotin-streptavidin labeling system.

### CD31 and pericyte staining

To examine changes in vascular density and the shape of blood vessels in aged kidneys, pericyte staining was performed in the presence of endothelial marker staining CD31 (PECAM-1). Rabbit anti-NG2 antibody (1:100; Millipore, Billerica, MA, USA) was incubated overnight at 4°C following biotinylated anti-rabbit antibody (1:500; Vector) incubation at room temperature for 1h. The signal was amplified by incubation with streptavidin-conjugated with Alexa Fluor 647 (1:100; Invitrogen, Grand Island, NY, USA) for 45 min. To prevent non-specific staining for the primary antibodies from the same species pre-incubation with anti-rabbit IgG Fab (1:25; Jackson ImmunoResearch Laboratories, West Grove, PA, USA) was followed by rabbit IgG Fab incubation (1:25; Jackson ImmunoResearch Laboratories). Rabbit anti-PDGFRß antibody (1:100; Abcam, Cambridge, MA, USA) was incubated with tissue sections overnight at 4°C. Secondary donkey anti-rabbit antibody conjugated with Alexa Fluor 488 (1:100, Invitrogen) was incubated for 1 h at room temperature. Rat anti-mouse CD31 (1:100; Dianova, Hamburg, Germany) was incubated overnight at 4°C following incubation with secondary anti-rat antibody conjugated with Alexa Fluor 594 (1:100 Invitrogen). Throughout the three antigen staining procedures, control staining based on omitting a primary antibody incubation (each of tested antibodies) was performed.

Staining of pericyte markers was quantified by taking 20 images of the cortex and medulla using 200x total magnification. Single color images and marker system embedded in the software to track individual cells were used to facilitate quantification. Vascular density was assessed based on the number of CD31-expressing cell per tubule by taking 20 images of the cortex and medulla at 200x total magnification. A number of CD31^+^ cells was divided by the number of tubules per field. Fluorescent imaging was performed using EVOS^®^FL Cell Imaging System (Life Technologies). Images were collected using confocal microscopy on a Leica DMI400B.

### Alpha smooth muscle actin and pericyte staining

Three antigen co-staining for pericyte markers (PDGFRß, NG2) and alpha smooth muscle actin (αSMA), which also may mark myofibroblast differentiation, was performed to investigate whether pericytes develop a pro-fibrotic phenotype during aging. Similarly as described above, NG2 antigen was detected using biotin-streptavidin system and labeled with Alexa Fluor 647. Anti-PDGFRβ antibody signal was visualized with goat anti-rabbit secondary antibody conjugated with Alexa Fluor 594. In the final step of the staining protocol, αSMA was (1:10.000; Sigma, Saint Louis, MI, USA) labeled using goat anti-mouse secondary antibody conjugated with Alexa Fluor 488 (Invitrogen).

The number of interstitial PDGFRß and NG2-expressing cells was collected. Additionally, the number of cells co-expressing αSMA in each population were calculated. αSMA positive cells in renal arterioles and vasa recta were considered as contractile vascular smooth muscle cells [72]. Cells expressing αSMA in peritubular and glomerular interstitial spaces were considered myofibroblasts [73]. Pericyte markers in cortex and medulla were quantified using 200x total magnification (20 images in cortex and in medulla). Single color images and marker system embedded in the software to track individual cells were used to facilitate quantification. Quantification was performed with Fluorescent imaging was performed using EVOS^®^FL Cell Imaging System (Life Technologies).

### Statistical analysis

One-way ANOVA was used to assess significance of Picrosirius Red and Collagen I staining. Two-tailed t-test was used to calculate significance for the number of pericytes, endothelial cells, and αSMA-expressing cells in young adult and aged group. Data were presented as means ± SEM (n=6). All data were analyzed in GraphPad Prism 5.0 (GraphPad Software, La Jolla, CA).
